# Metabolic and Immune Adaptations in Preterm Neonates at Early Postnatal Period: Integrated Analysis of Key Metabolites and Pathways

**DOI:** 10.1155/humu/9978047

**Published:** 2025-12-08

**Authors:** Xiaofan Li, Yue Gan, Lan Tan, Yuxi Lin, Pengxi Zhou, Jue Wang, Bing Yang, Quan Tang

**Affiliations:** ^1^ College of Life Sciences and Oceanography, Shenzhen University, Shenzhen, China, szu.edu.cn; ^2^ Shenzhen Institute for Drug Control, Shenzhen Key Laboratory of Drug Quality Standard Research, Shenzhen, China; ^3^ Department of Cell Biology, College of Basic Medical Sciences, Tianjin Medical University, Tianjin, China, tijmu.edu.cn; ^4^ Department of Public Health, International School, Krirk University, Bangkok, Thailand, krirk.ac.th; ^5^ Research Laboratory, Shenzhen Baoan Women′s and Children′s Hospital, Shenzhen, China

**Keywords:** full-term neonates, immunological adaptations, metabolomic profiling, postnatal adaptation, preterm neonates

## Abstract

**Objective:**

This study was aimed at clarifying the unique metabolic alterations in preterm neonates, distinct from full‐term neonates, between the first 24 and 48 h postnatally.

**Methods:**

A cohort of 60 preterm and 60 full‐term neonates was analyzed. The metabolomic profiles of plasma samples were determined using ultra performance liquid chromatography coupled with quadrupole time‐of‐flight mass spectrometry (UPLC‐QTOF‐MS). Multivariate statistical analyses were employed to discern metabolic differences. Multiple machine learning models were constructed to further select key metabolites. Spearman′s correlation analysis was performed to assess the correlation between neonatal immune cell subsets and key metabolites.

**Results:**

The study revealed 70 specific metabolic alterations in preterm neonates during the early postnatal period. Then, 32 of these metabolites were jointly selected by the Top 5 machine learning models, which exhibited high predictive performance with an AUC > 0.9. Subsequent analyses including multivariable linear regression and ROC curve revealed 12 key metabolites significantly associated with gestational age. Correlation analyses exposed significant associations between immune cells and these metabolites. Integrated pathway analysis identified 10 key metabolic pathways involved in preterm neonates. NMR‐based validation confirmed two of the 12 prioritized metabolites and six additional metabolites from the broader panel, supporting the robustness of our findings.

**Conclusion:**

Our findings provide insights into the metabolic and immune adaptation processes in preterm neonates during the early life stage. The correlations between immune cell subsets and the key metabolites highlight a potential effect of metabolism on immune adaptation in preterm neonates. These key metabolites and pathways could serve as potential biomarkers for early diagnosis and therapeutic strategies to enhance immune function and health outcomes in preterm infants.

## 1. Introduction

Preterm birth (< 37 weeks of gestation) occurs in approximately 10% of deliveries worldwide [1]. Prematurity and its associated complications are the leading direct cause of neonatal mortality [2], imposing a significant burden on healthcare systems as well as families.

The neonatal period is a critical and complex stage of human life, with metabolic processes facilitating rapid growth and healthy development. Neonates establish gluconeogenesis, modulate hepatic enzymes, and enhance lipolytic activity to stabilize blood glucose and meet energy demands after birth. Premature neonates exhibit distinct metabolic profiles that can affect their stress responses [3, 4], blood sugar level stability [5], and lipid metabolism [6]. These anomalies influence growth [7], immune function [8], and cognitive development [9], highlighting the distinct metabolic demands of preterm neonates.

Metabolic adaptation refers to the physiological process by which the body adjusts its metabolic rate and pathways in response to changes in nutritional status, energy demand, or pathological stress [10–12]. The metabolic adaptations in preterm infants differ significantly from those in term infants, contributing to increased risks of morbidity and mortality directly or indirectly. Therefore, early identification and understanding of these differences in metabolic adaptations are essential for optimizing clinical predictions and improving outcomes.

Studies have identified key metabolic changes in preterm infants, including alterations in amino acids, glucose metabolism, and energy pathways [13–15]. For instance, significant changes in amino acids (glycine and threonine) and tricarboxylic acid cycle metabolites (succinate and citrate) were observed early postnatally, correlating with postmenstrual age [16]. Urinary metabolite profiles in preterm infants show increased levels of betaine, glycine, and alanine, while taurine and myo‐inositol are decreased within the first 72 h of life, reflecting rapid metabolic adaptation to extrauterine life [17]. Additionally, levels of metabolic mediators like leptin, adiponectin, and insulin‐like growth factor 1 (IGF‐1) vary with maternal and neonatal factors, influencing the metabolic adaptation process [18].

Despite these promising advances, the clinical application of metabolomics remains limited. To our knowledge, this study represents the first investigation of metabolic adaptations in preterm neonates during the critical 24–48 h postnatal period. Notably, no prior research has studied the correlation between these transitional metabolites and immune cells during early life adaptation.

This study is aimed at identifying unique metabolic adaptation biomarkers in preterm infants during the 24–48 h postpartum, in comparison to full‐term infants, by using untargeted metabolomics with UPLC‐MS. A cohort of 60 preterm and 60 full‐term neonates was analyzed using multivariate statistical methods (PCA and OPLS‐DA), along with various machine learning models and further multivariate statistical analysis to refine biomarker selection. Additionally, the correlation between these key metabolites and neonatal immune cells was explored.

## 2. Materials and Methods

### 2.1. Study Population and Sample Collection

Eligible 120 infants, including 60 preterm neonates (28–37 weeks of gestation) and 60 full‐term neonates (38–42 weeks of gestation) born at the Shenzhen Bao’an Women′s and Children′s Hospital were included. The study was approved by the Ethics Committee of Shenzhen Baoan Women′s and Children′s Hospital with the ethical approval number LLSCHY‐2024‐01‐04‐03, and written informed consent was obtained from the legal guardians. All work was conducted in accordance with the Declaration of Helsinki (1964).

Infants with congenital malformations, genetic abnormalities, chromosomal disorders, inborn errors of metabolism, or severe congenital metabolic disorders were excluded from the study. To minimize confounding, only neonates with comparable postnatal age (24–48 h) at sampling and those receiving standardized feeding protocols were included.

Clinical data and immunocyte data were exported from the medical record system of our hospital. Plasma samples were collected and stored at −80°C.

### 2.2. Immune Cell Testing

Plasma samples were derived from blood samples after the completion of clinical routine hematological examinations, which were conducted by Sysmex XN‐9000. These routine hematological assays involve measuring the quantity and proportion of immune cells in the blood, including nucleated red blood cells (NRBCs), immature granulocytes (IGs), monocytes, lymphocytes, neutrophils, eosinophils, basophils, and so forth. The data were directly retrieved from the medical record system.

### 2.3. UPLC‐QTOF‐MS Analysis

Plasma samples were processed and analyzed based on the method described in reference [19] with modifications. Briefly, 100 *μ*L of thawed plasma was mixed with 10 *μ*L of internal standard (L‐2‐chlorophenylalanine) and precipitated with methanol–acetonitrile, 2:1, *v*/*v*. Then, 200 *μ*L of the supernatant was dried under nitrogen and reconstituted in 300 *μ*L of chilled methanol–water (1:4, *v*/*v*) for analysis. QC samples were prepared as previously reported [19] and interspersed every 10 experimental samples. Metabolite profiling was conducted using a Xevo G2‐XS QTOF spectrometer (Waters, Milford, United States) with an ACQUITY UPLC HSS T3 column at 45°C. The mobile phases were composed of 0.1% formic acid in water (A) and in acetonitrile (B). The gradient elution program was set as follows: 0–2 min, 5% B; 2–4 min, 5%–30% B; 4–8 min, 30%–50% B; 8–10 min, 50%–80% B; 10–14 min, 80%–100% B; and 14–15 min, 100% B. Mass spectrometry was operated in positive ESI+ mode (m/z 100–1500). Data were processed using Progenesis QI software for peak alignment, extraction, and metabolite identification.

### 2.4. Multivariate Statistical Analysis

The MS data acquired from Section 2.3 were refined by eliminating background ions and excluding features with a coefficient of variance (CV) of less than 50%. The curated data were then imported into SIMCA‐P 14.0 software (Umetrics, Umea, Sweden) for multivariate statistical analysis. Principal component analysis (PCA) was initially conducted to visualize the global distribution of samples. Orthogonal partial least squares discriminant analysis (OPLS‐DA) models were then constructed to delineate between‐group variations. The S‐plot and volcano plot methodologies were utilized to screen for differential metabolites, which were determined by three factors: VIP value, fold change (FC), and the probability value of significance test (*p*). One‐way analysis of variance (ANOVA) was applied for statistical significance testing, with a *p* value < 0.05.

### 2.5. Identification of Differential Metabolites

Following multivariate statistical analysis, the significantly differential features identified from the MS data were further characterized by matching their exact molecular weights and fragment ions with known compounds in established databases. The databases used for metabolite identification in this study included the Human Metabolome Database (HMDB), METLIN, Mass Bank, Kyoto Encyclopedia of Genes and Genomes (KEGG), LIPID Maps, and ChemSpider.

### 2.6. Machine Learning Model Construction and Receiver Operating Characteristic (ROC) Curve Analysis

A systematic optimization of parameters across various machine learning models was employed to enhance predictive capabilities using the SPSSPRO platform (https://www.spsspro.com). The unique differential metabolites of preterm infants at 24 and 48 h after birth served as dependent variables, while the postnatal time point was the independent variable in constructing the matrix of machine learning models. The dataset was partitioned into a training set (70%) and a testing set (30%). To ensure robust model evaluation, a threefold cross‐validation approach was employed, using the ROC curve, accuracy, recall, precision, *F*1 score, and area under the receiver operating characteristic curve (AUC) as parameters for model evaluation.

The ROC curve analysis of the refined metabolites with significant differences was conducted using MetaboAnalyst 4.0. The ROC curve was generated using the support vector machine (SVM) algorithm, and validation was performed using Monte Carlo cross‐validation (MCCV) with balanced subsampling. Metabolites with low reproducibility (RSD > 20*%*) were filtered out to eliminate baseline noise with the external AUROC percentage used as an indicator to evaluate model accuracy.

### 2.7. Statistical Analysis

The clinical data of neonates and their mothers were analyzed between preterm infants (P group) and full‐term infants (F group) using SPSS Statistics (IBM, NY, United States). The continuous variables were compared by univariate *t*‐tests, and categorical variables were analyzed using *χ* ^2^ tests. The features demonstrating significant variability through demographic analysis were extracted and multivariate analysis of variance (MANOVA) was conducted to assess the influence of these features on the coefficients of variation for differential metabolites. The relative impacts of each feature were quantified by the *F* values in a bar chart. Multivariate linear regression analysis was executed to correct confounding features affecting the concentration of the differential metabolites detected from UPLC‐MS spectra. The concentration of differential metabolites in neonates was set as the dependent variable, while the gestational age was considered the focal exposure factor. The other clinical attributes were incorporated as covariates.

The Shapiro–Wilk (SW) test was conducted using IBM SPSS Statistics software to assess the normality of the absolute counts and proportion of immune cells. Mann–Whitney *U* tests were performed to analyze the significance of differences among the groups subsequently. The Spearman′s correlation analysis was conducted to assess the correlation between neonatal immune cell subsets and key metabolites using Origin software.

### 2.8. Metabolic Pathway Enrichment

The MetaboAnalyst 6.0 cloud analysis platform was utilized to upload the information on significantly differential metabolites, such as compound names, HMDB numbers, and KEGG numbers. Subsequently, the hypergeometric test was selected as the enrichment method, the relative‐betweenness centrality was chosen as the topology analysis strategy, and the KEGG metabolic pathway database was selected as the pathway library. Finally, a KEGG‐related metabolic pathway bubble chart was constructed to visualize the enriched pathways.

### 2.9. NMR Analysis

The ^1^H NMR data were acquired on a Bruker AVANCE III 600 MHz NMR spectrometer equipped with a CPQCI cryoprobe using 200 *μ*L of neonatal plasma samples. The CPMG (Carr–Purcell–Meiboom–Gill) pulse sequence was selected for its effectiveness in enhancing the signal‐to‐noise ratio of the spectra. The parameters were optimized as follows: *T* = 298 K, NS = 64, DS = 4, AQ = 3.999 s, TD = 39,392, SW = 14 ppm, O1P = 4.705 ppm, and D1 = 4 s. The acquired 1H‐NMR data were processed using TOPSPIN 3.5 software (Bruker Biospin, Germany) and imported into MestReNova 14.0. The spectra were binned at intervals of 0.04 ppm, normalized to a total integral of 100, and the resulting data matrix was exported to an Excel file.

## 3. Results

### 3.1. Participant Characteristics

The demographic and clinical characteristics of the participating neonates and their mothers are summarized in Table 1. Among the maternal clinical features, the prevalence of prenatal hormone (dexamethasone) use was significantly higher in the preterm group compared to the full‐term group. Conversely, the incidence of premature rupture of membranes and the use of antibiotics was notably lower in the preterm cohort. Regarding neonatal characteristics, the average birth weight and birth length in the preterm group were significantly lower than those of the full‐term group. Furthermore, the preterm group exhibited increased rates of hyperbilirubinemia (31.7%) and hypokalemia (15%).

**Table 1:** Demographic and clinical characteristics of the participants.

	**Preterm (** **n** = 60 **)**	**Full-term (** **n** = 60 **)**	**p** **value**
Maternal characteristics			
Pregnancy diabetes, *n* (%)	21 (35%)	15 (25%)	0.232
Hypertensive, *n* (%)	0 (0%)	3 (5%)	0.242
Premature rupture of membranes, *n* (%)	27 (45%)	30 (50%)	< 0.001 ^∗∗∗^
Prenatal hormone (dexamethasone) use, *n* (%)	36 (60%)	0 (0%)	< 0.001 ^∗∗∗^
Antibiotic use, *n* (%)	15 (25%)	45 (75%)	< 0.001 ^∗∗∗^
Neonatal characteristics			
Gestational age at birth, mean (SD), weeks	35.7 (1.1)	39.4 (1.1)	< 0.001 ^∗∗∗^
Birth weight, mean (SD), g	2418 (428)	3107 (458)	< 0.001 ^∗∗∗^
Birth length, mean (SD), cm	46.2 (2.6)	49.5 (1.9)	< 0.001 ^∗∗∗^
Male, *n* (%)	32 (53.3%)	32 (53.3%)	1
Spontaneous delivery, *n* (%)	22 (36.7%)	28 (46.7%)	0.267
Cesarean section, *n* (%)	38 (63.3%)	32 (53.3%)	0.267
Low birth weight infants (< 2500 g), *n* (%)	34 (56.7%)	8 (13.3%)	< 0.001 ^∗∗∗^
ABO hemolytic disease, *n* (%)	5 (8.3%)	5 (8.3%)	1
Neonatal hyperbilirubinemia, *n* (%)	19 (31.7%)	2 (3.3%)	< 0.001 ^∗∗∗^
Neonatal jaundice, *n* (%)	38 (63.3%)	22 (36.7%)	0.003 ^∗∗^
Vitro fertilization, *n* (%)	10 (16.7%)	0 (0%)	< 0.001 ^∗∗∗^
Twin pregnancies, *n* (%)	18 (30%)	1 (1.7%)	< 0.001 ^∗∗∗^
Patent foramen ovale, *n* (%)	52 (86.7%)	52 (86.7%)	1
Hypocalcemia, *n* (%)	4 (6.7%)	0 (0%)	0.127
Hypokalemia, *n* (%)	9 (15%)	0 (0%)	0.006 ^∗∗^

*Note:* Values represent mean ± standard deviation or categorical values (%). *P*‐values were determined using univariate *t*‐tests for continuous variables and *χ* ^2^ tests for categorical variables.

^∗^ *p* < 0.05,  ^∗∗^ *p* < 0.01, and  ^∗∗∗^ *p* < 0.001.

### 3.2. Identification of Unique Metabolic Alterations in Preterm Neonates

Plasma samples of 60 preterm and 60 full‐term neonates at 24 and 48 h postbirth were collected and analyzed by UPLC‐MS for different metabolic alterations in preterm and term neonates.

PCA analysis revealed a tendency for differentiation in the metabolic profiles at 24 and 48 h for both preterm and full‐term neonates. OPLS‐DA further enhanced the separation between the P24 (preterm neonates at 24 h) and P48 (preterm neonates at 48 h) groups (Figure 1a), as well as between the F24 (full‐term neonates at 24 h) and F48 (full‐term neonates at 48 h) groups (Figure 1b). Permutation testing with 200 permutations was conducted to ensure the reliability and validity of the OPLS‐DA models. The Q2 (0.744) and R2 (0.959) values for the P24/P48 model, and the Q2 (0.614) and R2 (0.94) values for the F24/F48 model, indicated their optimal predictive ability.

Figure 1Multivariate analysis of differential features across cohorts. (a) OPLS‐DA scores plot (blue: preterm neonates at 48 h postpartum and green: preterm neonates at 24 h postpartum). (b) OPLS‐DA scores plot (blue: full‐term neonates at 24 h postpartum and green: full‐term neonates at 48 h postpartum). (c) Venn diagram showing the specific and overlapping features from the P‐24/48 group and P‐24/48 group (green circle: differential variables in preterm during 24 and 48 h postpartum and blue circle: differential variables in Full‐term during the same interval). (d) Volcano plot showing the differential variable levels between the P24 group and the P48 group. The *x* *x*‐axis represents the log2 value of the fold‐change of relative abundances, and the *y* *y*‐axis represents the statistical significance (*p* value) of the calculated fold‐change ratios (blue dots: significantly decreased variables and red dots: significantly increased variables).

**Figure:**
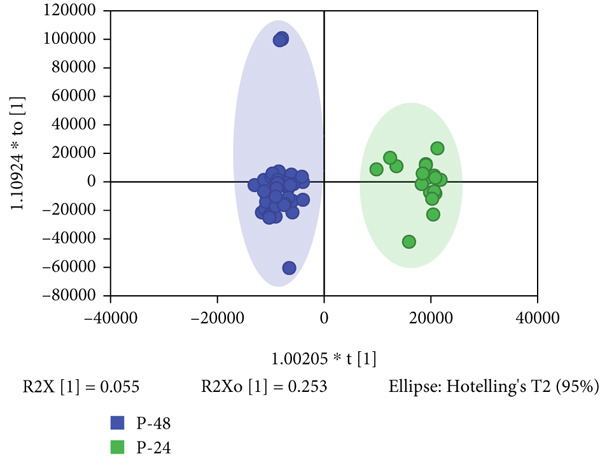
(a)

**Figure:**
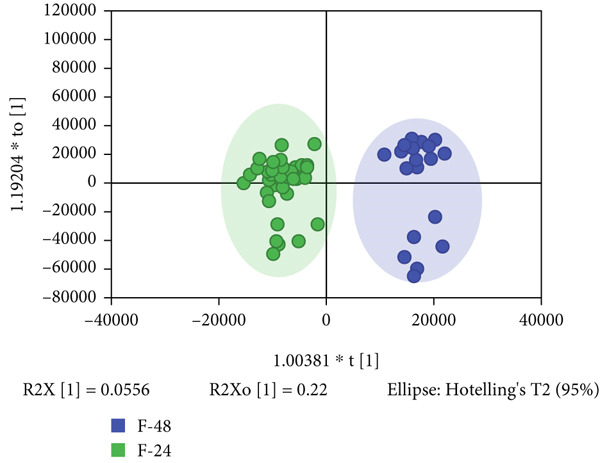
(b)

**Figure:**
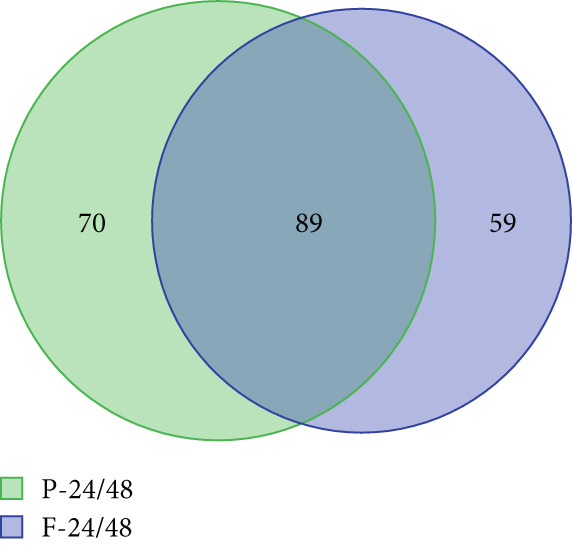
(c)

**Figure:**
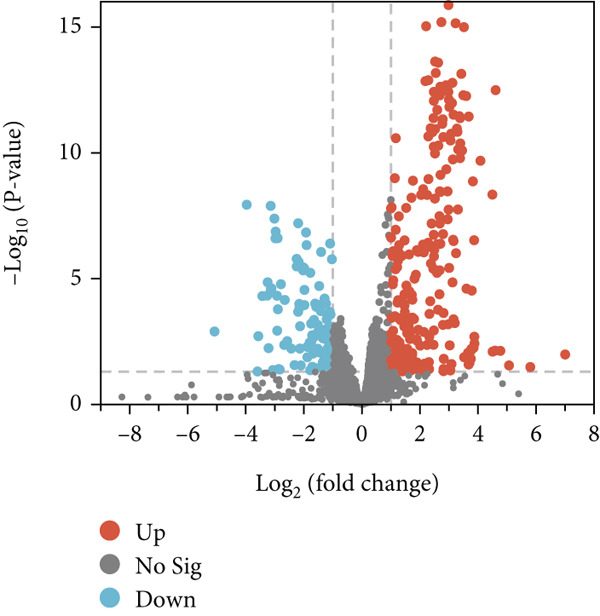
(d)

To further elucidate the differences in the differential variables obtained from the two distinct models, a Venn diagram was employed. Then, 89 differential variables were mutually present in both the preterm and full‐term groups, while 70 differential features were exclusive to the preterm group (Figure 1c), highlighting unique metabolic adaptation signatures that distinguish them from their full‐term counterparts. The volcano plot confirmed the 70 differential features in the preterm group based on the criteria of *p* value < 0.05 and |Log2FC| > 1 (Figure 1d).

### 3.3. Prediction of Unique Metabolic Adaption Signatures by Machine Learning Models

Seven machine learning models were built and optimized for better predictive power and generalizability by using the SPSSPRO platform.

Then, 70 specific features of the P24/P48 group acquired from the OPLS‐DA analysis (Figure 1c) served as the dependent variables, and the postpartum time was the independent variable for constructing the machine learning model matrix. The dataset was randomly divided into a training set (70%) and a test set (30%).

A threefold cross‐validation was conducted, using Accuracy, recall, precision, *F*1 score, and AUC to assess the predictive performance of the final model. As detailed in Table 2, the Top 5 models exhibit strong classification and predictive capabilities, with recall values (indicating the proportion of actual positives correctly identified by the model) exceeding 0.85 and AUC values (representing model performance across all possible classification thresholds) above 0.9, which are indicative of robust prediction as established in clinical research and omics analysis [20–25].

**Table 2:** Optimized machine learning models.

	**Accuracy**	**Recall**	**Precision**	**F**1	**AUC**
Random forest	0.944	0.944	0.948	0.942	1
CatBoost	0.889	0.889	0.904	0.879	1
ExtraTrees	0.944	0.944	0.949	0.944	0.968
Gradient boosting decision tree (GBDT)	0.889	0.889	0.963	0.913	0.941
LightGBM	0.944	0.944	0.949	0.944	0.922
Decision tree	0.889	0.889	0.889	0.889	0.862
*K*‐nearest neighbors (KNNs)	0.833	0.833	0.885	0.859	0.853

**Figure 2:**
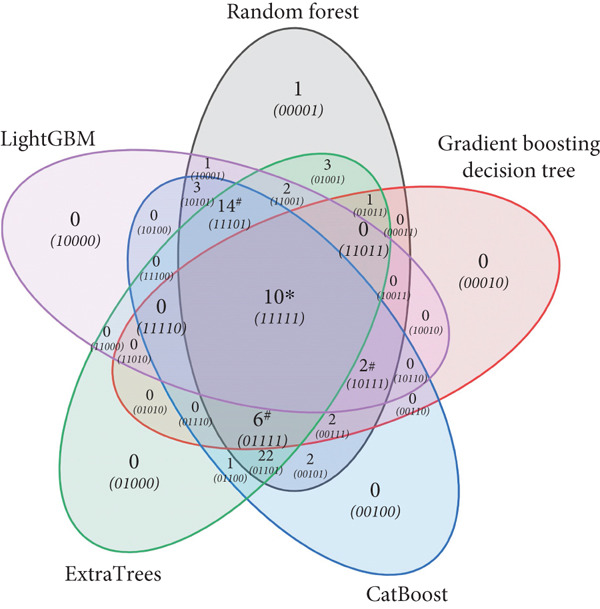
Differential features identification by the machine learning models. Venn diagram summarizing the results of common differential features predicted by the top five machine learning models: random forest, gradient boosting decision tree, CatBoost, ExtraTrees, and LightGBM. The diagram showed the number of differential features predicted by each model. A total of 32 differential features were jointly selected, including 10 ( ^∗^marked) shared by all five models, and an additional 22 (^#^marked) shared by four models.

To identify the common differential features predicted by the top five models, the 70 candidate features were analyzed based on the feature importance for each model algorithm, and a Venn diagram was plotted (Figure 2). A total of 32 differential features were jointly selected, including 10 ( ^∗^marked) shared by all five models, and an additional 22 (^#^marked) shared by four models. Detailed information on these metabolites is provided in Table S1.

### 3.4. Identification of 12 Metabolites Based on the Association Between the Metabolites and Clinical Data

Multivariable linear regression analysis was performed, with the abundance of 32 differential metabolites, as selected by machine learning models, treated as dependent variables. Gestational age was the focal independent variable, while multiple clinical factors (such as birth weight, length, hypokalemia, hypocalcemia, and patent foramen ovale) were included as explanatory variables in the model. As presented in Table 3, 12 of these compounds demonstrated significant associations with gestational age. The statistical information and trends for these 12 compounds in the P24 vs. P48 group are presented in Table 4. The results of multivariable linear regression analysis and statistical information for the total 32 metabolites were detailed in Tables S2 and S3.

**Table 3:** Multivariable linear regression analysis.

**No.**	**m**/**z**	**Metabolites**	***β*GA**	***P*GA**	***P*BW**	***P*BL**	***P*HypoK**	***P*HypoCa**	***P*PFO**
1	3.85_709.7909	Fuc*α*1‐2Gal*β*1‐4Glc*β*‐Sp	−0.474	0.002 ^∗∗^	0.596	0.045 ^∗^	0.248	0.125	0.743
2	3.85_827.9261	Glu Glu Thr Val Tyr	−0.46	0.003 ^∗∗^	0.466	0.071	0.337	0.158	0.676
3	5.92_296.2164	(11Z)‐8,18‐methano‐retinal	−0.454	0.004 ^∗∗^	0.437	0.14	0.95	0.142	0.639
4	5.31_371.1580	Asp Ala Pro Ser	−0.443	0.005 ^∗∗^	0.509	0.312	0.459	0.314	0.277
5	4.58_455.0462	L‐Glutamic acid 5‐phosphate	‐0.403	0.012 ^∗^	0.794	0.307	0.975	0.194	0.563
6	12.56_330.2747	MG(0:0/16:0/0:0)	0.394	0.012 ^∗^	0.319	0.145	0.286	0.349	0.38
7	1.94_191.0614	N‐Isobutyryl‐L‐cysteine	−0.393	0.01 ^∗^	0.432	0.977	0.438	0.008 ^∗∗^	0.357
8	13.65_387.2669	PG(16:0/20:3(5Z,8Z,11Z))	0.362	0.019 ^∗^	0.26	0.37	0.907	0.635	0.296
9	1.01_84.0459	7‐Methylguanine	−0.357	0.022 ^∗^	0.581	0.814	0.224	0.19	0.444
10	1.01_130.0522	1‐Methylguanine	−0.355	0.024 ^∗^	0.504	0.969	0.328	0.366	0.742
11	1.83_271.1007	Aspartyl‐histidine	−0.343	0.029 ^∗^	0.569	0.991	0.77	0.16	0.408
12	0.56_124.9802	3,5‐Dihydroxy‐4‐(sulfooxy)benzoic acid	−0.313	0.045 ^∗^	0.981	0.585	0.254	0.007 ^∗∗^	0.303

Abbreviations: BL, birth length; BW, birth weight; GA, gestational age; HypoCa, hypocalcemia; HypoK, hypokalemia; PFO, patent foramen ovale.

^∗^ *p* < 0.05 and  ^∗∗^ *p* < 0.01.

**Table 4:** Plasma differential metabolites from MS‐based metabolomics.

**No.**	**Metabolites**	**m**/**z**	**RT**	**VIP**	**p**	**FC**	**Trend (24/48 h)**
1	1‐Methylguanine	130.0522	1.01	4.2088	0.0183	2.6462	Up
2	3,5‐Dihydroxy‐4‐(sulfooxy)benzoic acid	124.9802	0.56	3.9794	0.0150	1.2154	Up
3	L‐Glutamic acid 5‐phosphate	455.0462	4.58	3.5861	0.0045	8.1539	Up
4	7‐Methylguanine	84.0459	1.01	2.3589	0.0442	3.2160	Up
5	(11Z)‐8,18‐methano‐retinal	296.2164	5.92	2.0232	0.0001	0.3277	Down
6	N‐Isobutyryl‐L‐cysteine	191.0614	1.94	1.7754	0.0263	1.4079	Up
7	Aspartyl‐Histidine	271.1007	1.83	1.7690	0.0259	4.6940	Up
8	Glu Glu Thr Val Tyr	827.9261	3.85	1.7606	0.0001	2.7687	Up
9	Fuc*α*1‐2Gal*β*1‐4Glc*β*‐Sp	709.7909	3.85	1.6871	0.0002	2.7951	Up
10	MG(0:0/16:0/0:0)	330.2747	12.56	1.5383	0.0217	0.3921	Down
11	PG(16:0/20:3(5Z,8Z,11Z))	387.2669	13.65	1.1255	0.0002	(0.3440)	Down
12	Asp Ala Pro Ser	371.158	5.31	1.0263	0.0000	(0.0182)	Down

*Note:* VIP > 1, *p* < 0.05, FC < 0.5, or FC > 2.

Abbreviations: FC, fold change; RT, retention time; VIP, variable important in the projection.

### 3.5. Validation of 12 Metabolites by ROC Curve and Longitudinal Boxplot Analyses

ROC curve analysis was performed to validate the discriminatory ability of the 12 key compounds. Among the models created with varying numbers of metabolites, the ROC curve model based on the 12 metabolites demonstrated the highest selectivity and sensitivity, with an AUC of 0.867 (95% confidence interval 0.715–0.986), indicating high classification accuracy between the two groups (Figure 3a). Based on the ROC analysis, the 12 compounds were identified as potential biomarkers distinguishing the unique metabolic adaptation differences in preterm neonates during the 24–48 h postpartum period, with an accuracy of 83.4% (Figure 3b). The average importance of each metabolite to the classification results is shown in Figure 3c, where higher scores indicate stronger discriminatory power of the metabolites.

Figure 3Differential metabolites validation by receiver operating characteristic (ROC) curve analysis. (a) Area under the receiver operating characteristic curve (AUC) in the discovery and validation of the differential metabolite model, based on its average performance in all MCCV studies. (b) Prediction accuracy of the linear SVM model with an increasing number of biomarkers (red dot: highest sensitivity of the biomarker model composed of 12 compounds). (c) Significance of the Top 12 selected metabolites from the linear support vector machine (SVM) model.

**Figure:**
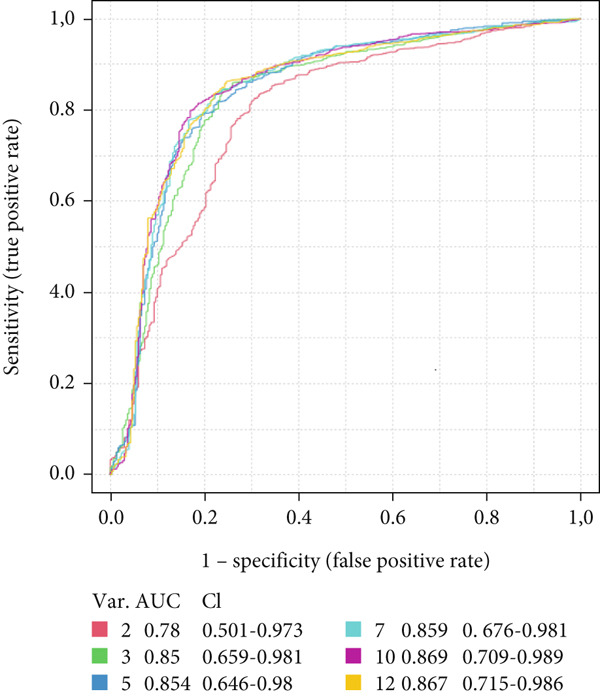
(a)

**Figure:**
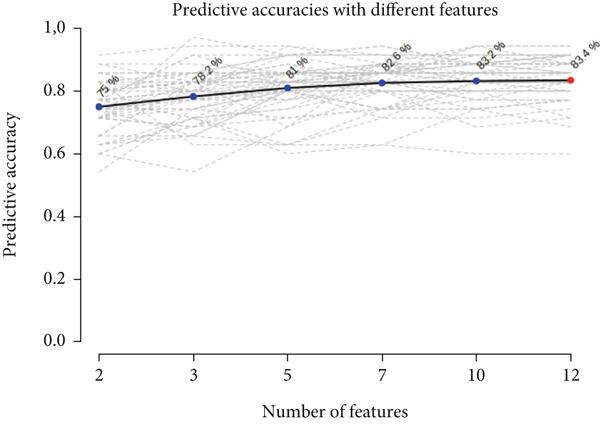
(b)

**Figure:**
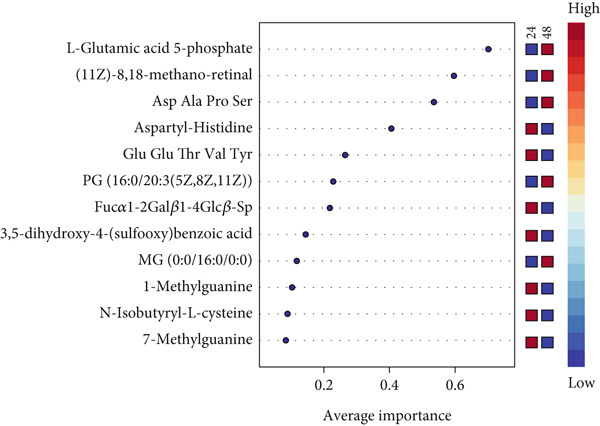
(c)

**Figure 4:**
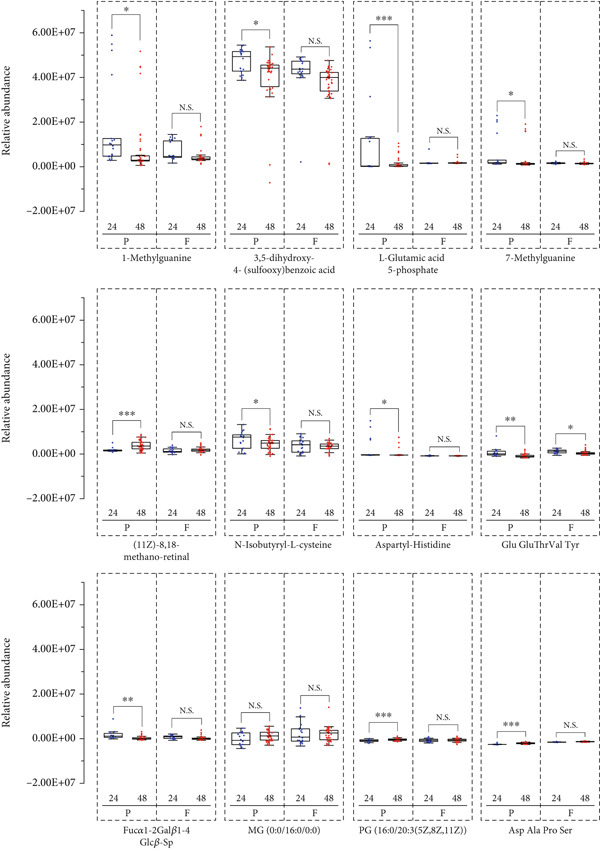
Boxplot of differential metabolite for preterm and full‐term neonates at 24 and 48 h postpartum. The intensity of differential metabolites identified by UPLC‐MS based on metabolomics (P, preterm; F, full term; blue: neonates at 24 h postpartum and red: neonates at 48 h postpartum).  ^∗^ *p* < 0.05,  ^∗∗^ *p* < 0.01, and  ^∗∗∗^ *p* < 0.001 using independent samples *t*‐tests.

Longitudinal boxplot analyses revealed distinct metabolic patterns between preterm and full‐term neonates during the critical 24–48 h postnatal period. As shown in Figure 4, preterm neonates showed significant declines (*p* < 0.05) in the levels of six metabolites: 1‐methylguanine (1‐mG), 3,5‐dihydroxy‐4‐(sulfooxy) benzoic acid, L‐glutamic acid 5‐phosphate, 7‐methylguanine (7‐mG), N‐isobutyryl‐L‐cysteine, and Fuc*α*1‐2Gal*β*1‐4Glc*β*‐Sp. Conversely, four metabolites increased over time in preterm cohorts: (11Z)‐8,18‐methano‐retinal, MG (0:0/16:0/0:0), PG (16:0/20:3(5Z,8Z,11Z)), and Asp Ala Pro Ser. Notably, full‐term neonates showed no significant changes (*p* > 0.05) in the levels of these metabolites during the same period.

### 3.6. Correlations Between Immune Cells and 12 Key Metabolites in Neonates

**Figure 5:**
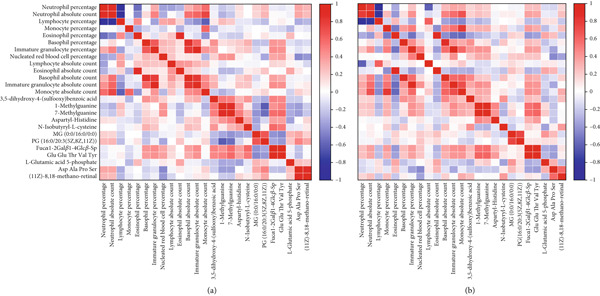
Correlation analysis between immune cells and key metabolites in neonates. (a) Heatmap based on Spearman correlation analysis in preterm neonates. (b) Heatmap based on Spearman correlation analysis in full‐term neonates. The longitudinal and the horizontal directions show the differential metabolites and the immune cells with significant differences in neonates at 24 to 48 h postpartum. The red color indicates a positive correlation and the blue color shows a negative correlation.  ^∗^ *p* < 0.05. The detailed correlation parameters were listed in Supplementary Table S4 and S5.

To further explore the effects of metabolic changes on immune cells in preterms (Figure 5a) and term (Figure 5b) neonates during the first 24–48 h postnatally, Spearman correlation analysis was used to examine the relationships between the absolute counts and percentages of immune cells and 12 key differential metabolites.

3,5‐Dihydroxy‐4‐(sulfooxy) benzoic acid was positively correlated with basophil percentage (*r* = 0.390, *p* = 0.002) and IG percentage (*r* = 0.370, *p* = 0.004). 1‐mG and 7‐mG showed a positive correlation with NRBC percentage, respectively (*r* = 0.443 and 0.458, *p* < 0.001).

Similarly, N‐isobutyryl‐L‐cysteine was positively correlated with IG percentage (*r* = 0.310, *p* < 0.05). PG(16:0/20:3(5Z,8Z,11Z)) showed a negative correlation with NRBC percentage (*r* = −0.386, *p* = 0.003).

Fuc*α*1‐2Gal*β*1‐4Glc*β*‐Sp exhibited multiple positive correlations, including with basophil percentage (*r* = 0.406, *p* = 0.001), IG percentage (*r* = 0.445, *p* < 0.001), and NRBC percentage (*r* = 0.341, *p* = 0.009).

Glu Glu Thr Val Tyr was similarly correlated with basophil percentage (*r* = 0.384, *p* = 0.003), IG percentage (*r* = 0.431, *p* < 0.001), and NRBC percentage (*r* = 0.331, *p* = 0.010). L‐Glutamic acid 5‐phosphate was positively correlated with eosinophil absolute count (*r* = 0.348, *p* = 0.007).

Asp Ala Pro Ser was positively correlated with neutrophil percentage (*r* = 0.347, *p* = 0.007) and negatively correlated with lymphocyte percentage (*r* = −0.304, *p* = 0.019) and NRBC percentage (*r* = −0.430, *p* < 0.001).

Similarly, (11Z)‐8,18‐methano‐retinal was positively correlated with neutrophil percentage (*r* = 0.282, *p* = 0.030) and negatively correlated with lymphocyte percentage (*r* = −0.262, *p* = 0.045) and NRBC percentage (*r* = −0.395, *p* = 0.002).

Several correlations observed between certain metabolites and immune cells in the preterm group were also detected in the full‐term group, such as the correlation between 3,5‐dihydroxy‐4‐(sulfooxy) benzoic acid with basophil and IG; 1‐mG and 7‐mG with NRBC; Fuc*α*1‐2Gal*β*1‐4Glc*β*‐Sp and Glu Glu Thr Val Tyr with basophil and IG as well as NRBC; L‐glutamic acid 5‐phosphate with eosinophil; and Asp Ala Pro Ser with NRBC. This observation suggested a potential conserved regulatory mechanism across different gestational ages, highlighting the importance of these metabolites in neonatal immune metabolism.

However, there were also unique correlations observed exclusively in either the preterm or full‐term group, suggesting potential differences in metabolic and immune regulatory patterns between the two groups during the early postnatal period. The detailed correlation data were provided in Table S4 for preterm neonates and Table S5 for term neonates.

### 3.7. Pathway Analysis of Preterm Neonates at 24 and 48 h Postpartum

**Figure 6:**
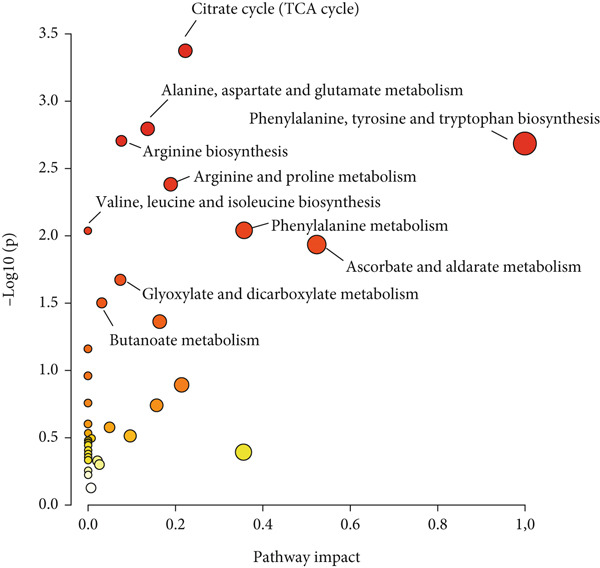
Bubble plot of metabolic pathway analysis related to differential metabolites in preterm neonates at 24 and 48 h postpartum. The *x*‐axis and the size of the bubble represent the impact factor of the pathway in the topological analysis. The *y*‐axis and the color of the bubble represent the *p* value of the enrichment analysis (displayed as −log10(*p*)). The color intensity reflects the significance of the *p* value and the degree of enrichment.

To elucidate the biological functions of the differential metabolites identified in preterm neonates, an integrated pathway analysis was conducted on 70 differential metabolites identified through UPLC‐QTOF‐MS platforms. The Top 10 pathways with the highest correlation were listed in Table S6. The topological impact factors and the *p* values from enrichment analysis are graphically represented in a bubble plot (Figure 6). Using the criterion of *p* < 0.01, seven metabolic pathways were identified as having a significant influence in preterm neonates at 24–48 h postpartum: the citrate cycle (TCA cycle), alanine, aspartate, and glutamate metabolism; arginine biosynthesis; phenylalanine, tyrosine, and tryptophan biosynthesis; arginine and proline metabolism; valine, leucine, and isoleucine biosynthesis; and phenylalanine metabolism.

### 3.8. Cross‐Platform Validation of Metabolic Biomarkers via NMR Spectroscopy

**Figure 7:**
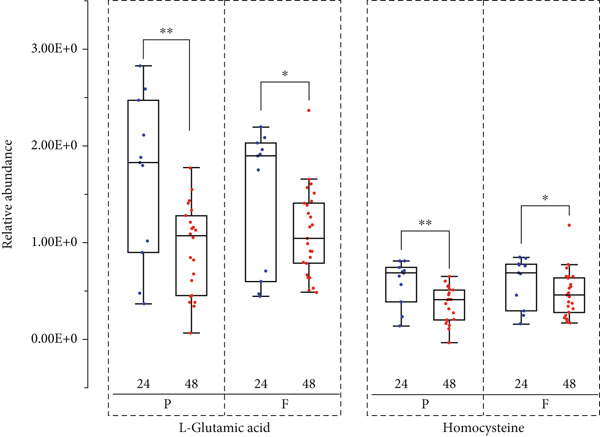
Boxplot of differential metabolites between preterm and full‐term neonates at 24 and 48 h postpartum. The relative abundance of selected metabolites was measured using NMR‐based metabolomics. (P, preterm; F, full term; blue: neonates at 24 h postpartum and red: neonates at 48 h postpartum).  ^∗^ *p* < 0.05,  ^∗∗^ *p* < 0.01, and  ^∗∗∗^ *p* < 0.001 using independent samples *t*‐tests.

Validation of potential biomarkers was performed using NMR‐based metabolomics to confirm their reproducibility and robustness across analytical platforms. From the 32 consensus metabolic biomarkers identified by top‐performing machine learning models, 12 were prioritized as key candidate metabolites based on their classification performance and clinical data. Among these 12 metabolites, two were successfully validated by NMR (Figure 7). Of the remaining 20 metabolites, six were also validated by NMR (Figure S2 and Table S7).

## 4. Discussion

In this study, 12 key metabolites were identified as distinct alterations in preterm neonates within the first 24–48 hours postpartum, using UPLC‐MS‐based metabolomics combined with machine learning validation. Additionally, correlations between these metabolites and neonatal immune cells were analyzed.

Retinal and its derivatives play pivotal roles in immune system regulation, particularly through their influence on immune cell differentiation and function. Retinoic acid derived from retinal dendritic cells modulates immune responses by influencing mucosal immunity, immunoglobulin production such as IgA, enhancing B cell activation markers such as CD38, and promoting the differentiation of regulatory T cells (Tregs) [26, 27]. (11Z)‐8,18‐methano‐retinal, a retinal derivative, binds to rhodopsin with comparable efficiency, suggesting its potential role in modulating visual processes while maintaining relevance to immune function [28]. Our study has revealed a statistically significant elevation of (11Z)‐8,18‐methano‐retinal levels in preterm cohorts within 24–48 h postpartum, a trend not observed in term infants. We also found a correlation between this compound and the neutrophil percentage in preterm neonates, suggesting that preterm neonates may exhibit heightened demand or altered metabolic handling of retinal derivatives during early postnatal adaptation. The observed increase could reflect compensatory mechanisms to stabilize retinal‐dependent pathways critical for both visual signaling and immune maturation. Our study reveals a positive correlation between retinal derivatives and neutrophil percentage. While current evidence lacks direct studies correlating serum retinal derivatives with neutrophil percentage in preterm neonates, the relationship between systemic inflammation, neutrophil activity, and retinal health in clinical observations like retinopathy of prematurity (ROP) suggests a potential indirect connection [29, 30]. Further research would be needed to explore this relationship explicitly.

Notably, the retinal has been identified as a potential biomarker for hepatocellular carcinoma (HCC). Retinal levels were significantly lower in HCC tumor tissues and serum samples compared to normal controls and cirrhotic patients [31]. This decrease may be due to disrupted retinol metabolism during HCC progression. Retinol and its metabolites are essential for cellular differentiation and tumor inhibition, with deficiency linked to immunodeficiency and increased carcinogenesis [32]. In liver cancer, loss of retinoids from hepatocytes and hepatic stellate cells promotes HCC development [33, 34]. Additionally, in bile duct ligation (BDL) models mimicking liver disease, retinal degeneration was observed, emphasizing the systemic impact of liver dysfunction on retinal health [35].

L‐Glutamate 5‐phosphate, a critical intermediate in the glutamate–glutamic acid metabolic axis, demonstrates profound functional linkages to immune cell proliferation and activation. [36] Dendritic cell‐derived glutamate can modulate T‐cell activation and cytokine secretion via glutamate receptors. [37] Glutamic acid, the precursor of glutamate, is also involved in the polarization of macrophages into M1 and M2 phenotypes [38]. Our findings reveal a significant decline in L‐glutamate 5‐phosphate levels within 24–48 h postpartum, correlating with eosinophil absolute counts and percentages in preterm infants. This suggests a potential regulatory role of glutamate metabolism in eosinophil maturation or recruitment. Although direct experimental evidence linking glutamate metabolism to eosinophils remains limited in current literature, its regulatory role can be inferred from its established involvement in immune cell metabolism and signaling networks [39, 40]. Given glutamate′s critical role in cellular energy metabolism, it is plausible that it contributes to eosinophil maturation and recruitment via metabolic pathways.

The metabolic products of tRNA degradation, 1‐mG and 7‐mG, have been reported to influence cellular properties, including adhesion and transformation potential [41], which are vital in immune responses and cancer progression. Specifically, 7‐mG has been shown to modulate the recruitment and activation of CD4^+^ T cells, mast cells, eosinophils, and natural killer T cells during liver ischemia‐reperfusion injury (LIRI) [42]. In cancer, 7‐mG has been implicated in the progression of various malignancies [43], particularly HCC. Studies have demonstrated that 7‐mG influences the tumor microenvironment, contributing to HCC development by altering cellular metabolism and immune regulation [44].

Our research reveals that both 1‐mG and 7‐mG levels significantly decrease in preterm neonates within 24–48 h postpartum. Interestingly, these compounds show a positive correlation with the percentage of NRBCs and a negative correlation with the absolute eosinophil count. This suggests that the depletion of tRNA modifications, specifically 1‐mG and 7‐mG, may disrupt immune cell proliferation critical for eosinophil maturation and stress‐adaptive erythropoiesis. Further investigation into how these compounds regulate immune cell activity in the early stages of life could provide valuable insights into their potential role in neonatal immune function.

Cross‐platform validation using NMR‐based metabolomics confirmed the robustness of biomarkers identified through UPLC‐MS and machine learning approaches. Two of the 12 metabolites were successfully validated by NMR, supporting their consistency across analytical platforms. L‐Glutamic acid 5‐phosphate is formed through the direct phosphorylation of glutamic acid and plays a central role in both proline biosynthesis and the urea cycle [45]. N‐Isobutyryl‐L‐cysteine and homocysteine are metabolically linked through sulfur amino acid metabolism. Homocysteine can be converted to cysteine, which serves as a precursor for isobutyryl cysteine [46]. Notably, six of the remaining 20 metabolites were also validated by NMR, further strengthening their potential utility for early diagnosis and therapeutic intervention. The lack of NMR validation for the remaining metabolites was likely due to the limited availability of neonatal plasma samples, which constrained the sample volume obtainable for analysis. As a result, the detection of low‐abundance metabolites remained challenging under limited samples. In addition, certain compounds, such as high‐molecular‐weight metabolites like peptides and fatty acids (e.g., Metabolite No. 7, 8, 10, 11, and 12 in Table 4), may lie outside the optimal detection range of NMR.

To the best of our knowledge, no studies have specifically investigated differential metabolites associated with metabolic adaptation in preterm neonates within the first 24–48 h postpartum. Moreover, no prior research has explored the correlation between these early‐life metabolic adaptations and immune cell populations. A recognized limitation of this work is the relatively small cohort size (*n* = 60 preterm and *n* = 60 full‐term), constrained by the challenges in obtaining paired plasma samples from preterm neonates at both 24 and 48 h postpartum.

## 5. Conclusions

The clinical significance of our study lies in the comprehensive insight into the unique metabolic and immune adaptations in preterm neonates during the critical early postnatal period, highlighting significant differences with potential implications for neonatal health and disease susceptibility. The identification of 12 key metabolites significantly associated with gestational age, along with their strong predictive performance (AUC > 0.9), underscores their potential as biomarkers for early prediction of complications in this vulnerable population. Moreover, the correlations between these metabolites and immune cell subsets underscore the potential impact of metabolic regulation on the immunity of preterm neonates. Specifically, two of the 12 prioritized candidate biomarkers and six additional metabolites from the broader panel of 32 consensus biomarkers were successfully validated by NMR spectroscopy. These key metabolites may be potential targets for strategies to enhance the immune function of preterm infants. The results underscore the necessity for further research on these metabolic differences and emphasize the need for the metabolic and immunological requirements of preterm neonates.

## Ethics Statement

We confirm that all experimental protocols were approved by the ethics committee of Shenzhen Bao’an Women′s and Children′s Hospital and the committee′s reference number is LLSCHY‐2024‐01‐04‐03, and the written informed consent was obtained from the legal guardians. All works were conducted in accordance with the Declaration of Helsinki (1964).

## Disclosure

All authors have read and agreed to the published version of the manuscript.

## Conflicts of Interest

The authors declare no conflicts of interest.

## Author Contributions

The contributions of the respective authors are as follows: X.L. contributed to the experimental design, funding acquisition, and original manuscript drafting; Y.G. contributed to statistical processing and machine learning models; L.T. and P.Z. contributed to the MS spectrometry analysis; Y.L. contributed to the correlation analysis; J.W. contributed to MS spectrometry; B.Y. contributed to the review of the manuscript and polishing the language; Q.T. contributed to the establishment of the clinical cohort, funding acquisition, and manuscript revision.

## Funding

This work was supported by the Science and Technology Project of Shenzhen City, Shenzhen Bureau of Science, Technology, and Information, 20231121182401001; National Natural Science Foundation of China, 10.13039/501100001809, 82201897; Science, Technology and Innovation Commission of Shenzhen Municipality, 10.13039/501100010877, JCYJ20210324130005013; Health and Medical Scientific Research Project of Shenzhen Bao’an Medical Association, BAYXH2024027; and Science and Technology Project of Shenzhen City, JCYJ20190808114415068.

## Supporting information


**Supplementary Information** Additional supporting information can be found online in the Supporting Information section. Table S1: Common differential features predicted by the Top 5 machine learning models. Table S2: Multivariable linear regression analysis. Table S3: Plasma differential metabolites from MS‐based metabolomics. Table S4: Spearman correlation analysis of the key differential metabolites and immune cell profiles in preterm neonates. Table S5: Spearman correlation analysis of the key differential metabolites and immune cell profiles in full‐term neonates. Table S6: Pathway analysis of preterm neonates at 24 and 48 h postpartum. Table S7: Metabolites validated by NMR spectroscopy. Figure S1: Boxplot of six validated metabolites between preterm and full‐term neonates at 24 and 48 h postpartum. The intensity of these metabolites was determined using UPLC‐MS‐based metabolomics. Figure S2: Boxplot of six validated metabolites between preterm and full‐term neonates at 24 and 48 h postpartum. The relative abundance of selected metabolites was measured using NMR‐based metabolomics.

## Data Availability

The datasets generated/analyzed for this study are not publicly available but are available from the corresponding author upon reasonable request.
