# Supplementary material for: Metabolic and Immune Adaptations in Preterm Neonates at Early Postnatal Period: Integrated Analysis of Key Metabolites and Pathways
Source: Hum Mutat. 2025 Dec 8;2025:9978047. doi: 10.1155/humu/9978047 (PMC12714169; doi:10.1155/humu/9978047)
Supplement: Supplementary file 1 — Supplementary Information Additional supporting information can be found online in the Supporting Information section. Table S1: Common differential features predicted by the Top 5 machine learning models. Table S2: Multivariable linear regression analysis. Table S3: Plasma differential metabolites from MS‐based metabolomics. Table S4: Spearman correlation analysis of the key differential metabolites and immune cell profiles in preterm neonates. Table S5: Spearman correlation analysis of the key differential metabolites and immune cell profiles in full‐term neonates. Table S6: Pathway analysis of preterm neonates at 24 and 48 h postpartum. Table S7: Metabolites validated by NMR spectroscopy. Figure S1: Boxplot of six validated metabolites between preterm and full‐term neonates at 24 and 48 h postpartum. The intensity of these metabolites was determined using UPLC‐MS‐based metabolomics. Figure S2: Boxplot of six validated metabolites between preterm and full‐term neonates at 24 and 48 h postpartum. The relative abundance of selected metabolites was measured using NMR‐based metabolomics. [file HUMU-2025-9978047-s001.docx]

Supplementary Material

# Supplementary Data

Supplementary Table S1. Common differential features predicted by the top five Machine Learning Models

Supplementary Table S2. Multivariable linear regression analysis

Supplementary Table S3. Plasma Differential Metabolites from MS based metabolomics

Supplementary Table S4. Spearman Correlation analysis of the key differential metabolites and immune cell profiles in preterm neonates

Supplementary Table S5. Spearman Correlation analysis of the key differential metabolites and immune cell profiles in full-term neonates

Supplementary Table S6. Pathway Analysis of Preterm Neonates at 24 and 48 h. Postpartum

Supplementary Table S7. Metabolites validated by NMR Spectroscopy

Supplementary Figure S1. Boxplot of six validated metabolites between preterm and full-term neonates at 24 h and 48 h postpartum. The intensity of these metabolites was determined using UPLC-MS based metabolomics.

Supplementary Figure S2. Boxplot of six validated metabolites between preterm and full-term neonates at 24 h and 48 h postpartum. The relative abundance of selected metabolites was measured using NMR-based metabolomics.

# Supplementary Tables

Supplementary Table S1. Common differential features predicted by the top five Machine Learning Models

| m/z | Metabolites | Model |
| --- | --- | --- |
| 4.68_631.3478 | 19-Hydroxydeacetylnomilinic acid 17-beta-D-glucopyranoside | LGBM, ET, CB, GBDT, RT |
| 1.38_218.1414 | Benzyl butyl phthalate | LGBM, ET, CB, GBDT, RT |
| 5.31_371.1580 | Asp Ala Pro Ser | LGBM, ET, CB, GBDT, RT |
| 0.63_258.9061 | Choline | LGBM, ET, CB, GBDT, RT |
| 1.01_130.0522 | 1-Methylguanine | LGBM, ET, CB, GBDT, RT |
| 1.01_84.0459 | 7-Methylguanine | LGBM, ET, CB, GBDT, RT |
| 5.92_395.2233 | beta-Bixin | LGBM, ET, CB, GBDT, RT |
| 12.56_330.2747 | MG(0:0/16:0/0:0) | LGBM, ET, CB, GBDT, RT |
| 2.06_166.0882 | Phenylalanine | LGBM, ET, CB, GBDT, RT |
| 1.01_229.9784 | N-(3,4-Dichlorophenyl)-malonamate | LGBM, ET, CB, GBDT, RT |
| 3.85_709.7909 | Fucα1-2Galβ1-4Glcβ-Sp | LGBM, ET, CB, RT |
| 9.16_810.2727 | UDP-3-(3R-hydroxy-tetradecanoyl)-alphaD-glucosamine | LGBM, ET, CB, RT |
| 1.94_191.0614 | N-Isobutyryl-L-cysteine | LGBM, ET, CB, RT |
| 3.85_827.9261 | Glu Glu Thr Val Tyr | LGBM, ET, CB, RT |
| 0.55_255.9458 | Uracil, 2TMS derivative | LGBM, ET, CB, RT |
| 9.14_843.3887 | latrunculin a | LGBM, ET, CB, RT |
| 12.54_780.3017 | Estradiol monobenzoate | LGBM, ET, CB, RT |
| 3.55_163.0655 | Histidylproline diketopiperazine | LGBM, ET, CB, RT |
| 13.65_387.2669 | PG(16:0/20:3(5Z,8Z,11Z)) | LGBM, ET, CB, RT |
| 6.03_333.1490 | Cyproconazole | LGBM, ET, CB, RT |
| 0.56_256.9715 | 2-Hydroxy-2-methylbutyric acid | LGBM, ET, CB, RT |
| 6.72_462.2706 | N-Eicosapentaenoyl Histidine | LGBM, ET, CB, RT |
| 9.14_574.1587 | Viniferal | LGBM, ET, CB, RT |
| 0.56_198.9415 | 5-N-Acetyl-beta-D-neuraminic acid | LGBM, ET, CB, RT |
| 3.54_170.0629 | L-Tryptophan | LGBM, CB, GBDT, RT |
| 11.37_482.3365 | Fumaric acid, 2,3,4,6-tetrachlorophenyl undecyl ester | LGBM, CB, GBDT, RT |
| 5.92_296.2164 | (11Z)-8,18-methano-retinal | ET, CB, GBDT, RT |
| 4.58_455.0462 | L-Glutamic acid 5-phosphate | ET, CB, GBDT, RT |
| 0.56_124.9802 | 3,5-dihydroxy-4-(sulfooxy)benzoic acid | ET, CB, GBDT, RT |
| 0.81_215.0183 | Citric acid | ET, CB, GBDT, RT |
| 1.83_271.1007 | Aspartyl-Histidine | ET, CB, GBDT, RT |
| 1.01_441.0504 | 17-beta-Estradiol glucuronide | ET, CB, GBDT, RT |

LGBM: LightGBM, ET: ExtraTrees, CB: CatBoost, GBDT: Gradient Boosting Decision Tree, RT: Random Forest.

Supplementary Table S2. Multivariable linear regression analysis

| **No.** | m/z | Metabolites | βGA | *P*GA | *P*BW | *P*BL | *P*HypoK | *P*HypoCa | *P*PFO |
| --- | --- | --- | --- | --- | --- | --- | --- | --- | --- |
| **1** | 3.85_709.7909 | Fucα1-2Galβ1-4Glcβ-Sp | -0.474 | 0.002** | 0.596 | 0.045* | 0.248 | 0.125 | 0.743 |
| **2** | 3.85_827.9261 | Glu Glu Thr Val Tyr | -0.46 | 0.003** | 0.466 | 0.071 | 0.337 | 0.158 | 0.676 |
| **3** | 5.92_296.2164 | (11Z)-8,18-methano-retinal | -0.454 | 0.004** | 0.437 | 0.14 | 0.95 | 0.142 | 0.639 |
| **4** | 5.31_371.1580 | Asp Ala Pro Ser | -0.443 | 0.005** | 0.509 | 0.312 | 0.459 | 0.314 | 0.277 |
| **5** | 4.58_455.0462 | L-Glutamic acid 5-phosphate | -0.403 | 0.012* | 0.794 | 0.307 | 0.975 | 0.194 | 0.563 |
| **6** | 12.56_330.2747 | MG(0:0/16:0/0:0) | 0.394 | 0.012* | 0.319 | 0.145 | 0.286 | 0.349 | 0.38 |
| **7** | 1.94_191.0614 | N-Isobutyryl-L-cysteine | -0.393 | 0.01* | 0.432 | 0.977 | 0.438 | 0.008** | 0.357 |
| **8** | 13.65_387.2669 | PG(16:0/20:3(5Z,8Z,11Z)) | 0.362 | 0.019* | 0.26 | 0.37 | 0.907 | 0.635 | 0.296 |
| **9** | 1.01_84.0459 | 7-Methylguanine | -0.357 | 0.022* | 0.581 | 0.814 | 0.224 | 0.19 | 0.444 |
| **10** | 1.01_130.0522 | 1-Methylguanine | -0.355 | 0.024* | 0.504 | 0.969 | 0.328 | 0.366 | 0.742 |
| **11** | 1.83_271.1007 | Aspartyl-Histidine | -0.343 | 0.029* | 0.569 | 0.991 | 0.77 | 0.16 | 0.408 |
| **12** | 0.56_124.9802 | 3,5-dihydroxy-4-(sulfooxy)benzoic acid | -0.313 | 0.045* | 0.981 | 0.585 | 0.254 | 0.007** | 0.303 |
| **13** | 1.01_441.0504 | 17-beta-Estradiol glucuronide | -0.306 | 0.053 | 0.41 | 0.856 | 0.311 | 0.389 | 0.664 |
| **14** | 11.37_482.3365 | Fumaric acid, 2,3,4,6-tetrachlorophenyl undecyl ester | 0.271 | 0.079 | 0.221 | 0.415 | 0.381 | 0.174 | 0.765 |
| **15** | 1.38_218.1414 | Benzyl butyl phthalate | -0.256 | 0.1 | 0.345 | 0.855 | 0.054 | 0.095 | 0.437 |
| **16** | 0.56_256.9715 | 2-Hydroxy-2-methylbutyric acid | -0.254 | 0.112 | 0.496 | 0.71 | 0.874 | 0.457 | 0.14 |
| **17** | 1.01_229.9784 | N-(3,4-Dichlorophenyl)-malonamate | -0.252 | 0.117 | 0.419 | 0.697 | 0.656 | 0.774 | 0.734 |
| **18** | 0.81_215.0183 | Citric acid | -0.202 | 0.21 | 0.798 | 0.506 | 0.778 | 0.091 | 0.352 |
| **19** | 3.54_170.0629 | L-Tryptophan | -0.197 | 0.206 | 0.038* | 0.36 | 0.505 | 0.021* | 0.437 |
| **20** | 3.55_163.0655 | Histidylproline diketopiperazine | -0.194 | 0.214 | 0.062 | 0.198 | 0.76 | 0.007** | 0.657 |
| **21** | 0.56_198.9415 | 5-N-Acetyl-beta-D-neuraminic acid | -0.18 | 0.262 | 0.56 | 0.629 | 0.806 | 0.496 | 0.097 |
| **22** | 2.06_166.0882 | Phenylalanine | -0.126 | 0.432 | 0.257 | 0.225 | 0.956 | 0.791 | 0.325 |
| **23** | 5.92_395.2233 | beta-Bixin | -0.122 | 0.445 | 0.24 | 0.946 | 0.791 | 0.164 | 0.444 |
| **24** | 6.72_462.2706 | N-Eicosapentaenoyl Histidine | 0.117 | 0.468 | 0.2 | 0.534 | 0.784 | 0.426 | 0.988 |
| **25** | 0.63_258.9061 | Choline | -0.089 | 0.571 | 0.997 | 0.856 | 0.147 | 0.023* | 0.174 |
| **26** | 9.14_843.3887 | latrunculin a | 0.083 | 0.61 | 0.565 | 0.676 | 0.867 | 0.716 | 0.839 |
| **27** | 0.55_255.9458 | Uracil, 2TMS derivative | 0.075 | 0.637 | 0.382 | 0.364 | 0.32 | 0.113 | 0.658 |
| **28** | 4.68_631.3478 | 19-Hydroxydeacetylnomilinic acid 17-beta-D-glucopyranoside | -0.067 | 0.679 | 0.931 | 0.636 | 0.941 | 0.82 | 0.137 |
| **29** | 6.03_333.1490 | Cyproconazole | -0.055 | 0.729 | 0.98 | 0.514 | 0.586 | 0.359 | 0.037* |
| **30** | 9.16_810.2727 | UDP-3-(3R-hydroxy-tetradecanoyl)-alphaD-glucosamine | 0.047 | 0.763 | 0.219 | 0.244 | 0.013* | 0.213 | 0.222 |
| **31** | 9.14_574.1587 | Viniferal | 0.037 | 0.819 | 0.571 | 0.659 | 0.895 | 0.347 | 0.798 |
| **32** | 12.54_780.3017 | Estradiol monobenzoate | 0.036 | 0.823 | 0.947 | 0.737 | 0.429 | 0.772 | 0.814 |

GA: gestational age, BW: birth weight, BL: birth length, HypoK: hypokalemia, HypoCa: hypocalcemia, PFO: patent foramen ovale. Significance * indicates *P* < 0.05, ** indicates *P* < 0.01.

Supplementary Table S3. Plasma Differential Metabolites from MS based metabolomics

| **No.** | Metabolites | m/z | RT | VIP | *P* | FC | Trend  (24 h/48 h) |
| --- | --- | --- | --- | --- | --- | --- | --- |
| **1** | Viniferal | 574.1587 | 9.14 | 8.9170 | 0.0015 | 2.9534 | up |
| **2** | 1-Methylguanine | 130.0522 | 1.01 | 4.2088 | 0.0183 | 2.6462 | up |
| **3** | 3,5-dihydroxy-4-(sulfooxy)benzoic acid | 124.9802 | 0.56 | 3.9794 | 0.0150 | 1.2154 | up |
| **4** | L-Glutamic acid 5-phosphate | 455.0462 | 4.58 | 3.5861 | 0.0045 | 8.1539 | up |
| **5** | 2-Hydroxy-2-methylbutyric acid | 256.9715 | 0.56 | 3.3551 | 0.0016 | 1.4231 | up |
| **6** | Histidylproline diketopiperazine | 163.0655 | 3.55 | 3.2149 | 0.0102 | 1.2537 | up |
| **7** | Phenylalanine | 166.0882 | 2.06 | 2.6602 | 0.0212 | 1.2029 | up |
| **8** | 7-Methylguanine | 84.0459 | 1.01 | 2.3589 | 0.0442 | 3.2160 | up |
| **9** | Pyrazolo[1,5-a]pyrimidine | 780.3017 | 12.54 | 2.2862 | 0.0038 | 14.7577 | up |
| **10** | 5-N-Acetyl-beta-D-neuraminic acid | 198.9415 | 0.56 | 2.2031 | 0.0043 | 1.4210 | up |
| **11** | PA(15:0/14:1(9Z)) | 395.2233 | 5.92 | 2.0918 | 0.0000 | 0.2461 | down |
| **12** | (11Z)-8,18-methano-retinal | 296.2164 | 5.92 | 2.0232 | 0.0001 | 0.3277 | down |
| **13** | N-Isobutyryl-L-cysteine | 191.0614 | 1.94 | 1.7754 | 0.0263 | 1.4079 | up |
| **14** | Aspartyl-Histidine | 271.1007 | 1.83 | 1.7690 | 0.0259 | 4.6940 | up |
| **15** | Glu Glu Thr Val Tyr | 827.9261 | 3.85 | 1.7606 | 0.0001 | 2.7687 | up |
| **16** | Fucα1-2Galβ1-4Glcβ-Sp | 709.7909 | 3.85 | 1.6871 | 0.0002 | 2.7951 | up |
| **17** | Benzyl butyl phthalate | 218.1414 | 1.38 | 1.6112 | 0.0001 | 2.3449 | up |
| **18** | MG(0:0/16:0/0:0) | 330.2747 | 12.56 | 1.5383 | 0.0217 | 0.3921 | down |
| **19** | Turosteride | 482.3365 | 11.37 | 1.4964 | 0.0023 | 0.4804 | down |
| **20** | N-(3,4-Dichlorophenyl)-malonamate | 229.9784 | 1.01 | 1.3770 | 0.0051 | 2.5262 | up |
| **21** | N-Eicosapentaenoyl Histidine | 462.2706 | 6.72 | 1.3664 | 0.0126 | 1.7005 | up |
| **22** | 2-(Methylthiomethyl)furan | 170.0629 | 3.54 | 1.3395 | 0.0020 | 1.3212 | up |
| **23** | latrunculin a | 843.3887 | 9.14 | 1.2841 | 0.0003 | 5.0275 | up |
| **24** | Choline | 258.9061 | 0.63 | 1.2654 | 0.0201 | 1.2160 | up |
| **25** | 17-beta-Estradiol glucuronide | 441.0504 | 1.01 | 1.2478 | 0.0481 | 2.6456 | up |
| **26** | 3,5-Diiodothyropropionic acid | 255.9458 | 0.55 | 1.1649 | 0.0054 | 1.3426 | up |
| **27** | Citric acid | 215.0183 | 0.81 | 1.1424 | 0.0000 | 1.5745 | up |
| **28** | PG(16:0/20:3(5Z,8Z,11Z)) | 387.2669 | 13.65 | 1.1255 | 0.0002 | (0.3440) | down |
| **29** | UDP-3-(3R-hydroxy-tetradecanoyl)-alphaD-glucosamine | 810.2727 | 9.16 | 1.0753 | 0.0397 | 1.6916 | up |
| **30** | N-Succinyl-ala-ala-pro-phe-p-nitroanilide | 631.3478 | 4.68 | 1.0414 | 0.0000 | 5.1635 | up |
| **31** | Asp Ala Pro Ser | 371.158 | 5.31 | 1.0263 | 0.0000 | (0.0182) | down |
| **32** | Cyproconazole | 333.149 | 6.03 | 1.0206 | 0.0071 | 0.4768 | down |

RT: Retention Time, VIP: Variable Importance in the Projection, FC: Fold change, VIP>1, *P*<0.05, FC<0.5 or FC >2

Supplementary Table S4. Spearman Correlation analysis of the key differential metabolites and immune cell profiles in preterm neonates

| Metabolite | Parameter | Neutrophil Percentage | Neutrophil Absolute Count | Lymphocyte Percentage | Monocyte Percentage | Eosinophil Percentage | Basophil Percentage |
| --- | --- | --- | --- | --- | --- | --- | --- |
| 3,5-dihydroxy-4-(sulfooxy)benzoic acid | r | -0.04732 | -0.02046 | 0.06152 | -0.2773* | 0.01667 | 0.39039* |
|  | *p*-value | 0.72194 | 0.87778 | 0.64346 | 0.03348 | 0.90029 | 0.00224 |
| 1-Methylguanine | r | -0.19055 | -0.27981* | 0.25505 | -0.08919 | -0.1393 | 0.11956 |
|  | *p*-value | 0.14828 | 0.03184 | 0.05123 | 0.50174 | 0.29268 | 0.36707 |
| 7-Methylguanine | r | -0.1276 | -0.19676 | 0.18783 | -0.07741 | -0.18372 | 0.16163 |
|  | *p*-value | 0.33551 | 0.13527 | 0.15427 | 0.56007 | 0.16365 | 0.22133 |
| Aspartyl-Histidine | r | 0.11699 | 0.10067 | -0.07677 | -0.11085 | -0.19858 | 0.2134 |
|  | *p*-value | 0.37755 | 0.44805 | 0.5633 | 0.40325 | 0.13163 | 0.10462 |
| N-Isobutyryl-L-cysteine | r | 0.01824 | 0.02648 | -0.09489 | -0.05271 | 0.07021 | 0.29487* |
|  | *p*-value | 0.89096 | 0.84222 | 0.47466 | 0.69176 | 0.59723 | 0.02338 |
| MG(0:0/16:0/0:0) | r | 0.18827 | 0.16114 | -0.15732 | 0.00471 | 0.00833 | -0.21391 |
|  | *p*-value | 0.15329 | 0.22276 | 0.23406 | 0.97178 | 0.95005 | 0.10378 |
| PG(16:0/20:3(5Z,8Z,11Z)) | r | 0.23161 | 0.24018 | -0.22229 | 0.03224 | 0.10767 | -0.19851 |
|  | *p*-value | 0.07755 | 0.0669 | 0.09062 | 8.08E-01 | 0.41698 | 0.13175 |
| Fucα1-2Galβ1-4Glcβ-Sp | r | -0.05009 | 0.02811 | 0.02785 | -0.14283 | -0.03076 | 0.40647* |
|  | *p*-value | 0.70634 | 0.83261 | 0.83414 | 2.81E-01 | 0.81709 | 0.0014 |
| Glu Glu Thr Val Tyr | r | -0.06669 | -0.00342 | 0.05594 | -0.15955 | -0.04161 | 0.38385* |
|  | *p*-value | 0.61575 | 0.9795 | 0.6739 | 2.27E-01 | 0.75435 | 0.00269 |
| L-Glutamic acid 5-phosphate | r | -0.06582 | -0.05088 | -0.01037 | -0.23705 | 0.27694* | -0.11503 |
|  | *p*-value | 0.62041 | 0.70195 | 0.93784 | 7.07E-02 | 0.03372 | 0.38563 |
| Asp Ala Pro Ser | r | 0.3467* | 0.3256* | -0.30444* | -0.07609 | 0.0052 | -0.15103 |
|  | *p*-value | 0.00714 | 0.01185 | 0.01906 | 0.56677 | 0.96879 | 0.25352 |
| (11Z)-8,18-methano-retinal | r | 0.2822* | 0.25129 | -0.26177* | -0.09395 | -0.000848 | -0.10935 |
|  | *p*-value | 0.03035 | 0.05488 | 0.0452 | 0.47907 | 0.99491 | 0.40969 |

| Metabolite | Parameter | Immature Granulocyte Percentage | Nucleated Red Blood Cell Percentage | Lymphocyte Absolute Count | Eosinophil Absolute Count | Basophil Absolute Count | Immature Granulocyte Absolute Count | Monocyte Absolute Count |
| --- | --- | --- | --- | --- | --- | --- | --- | --- |
| 3,5-dihydroxy-4-(sulfooxy)benzoic acid | r | 0.37029* | 0.22904 | 0.04489 | -0.02813 | 0.24732 | 0.30103* | -0.1904 |
|  | *p*-value | 0.00422 | 0.08373 | 0.73566 | 0.83251 | 0.05896 | 0.02288 | 0.14861 |
| 1-Methylguanine | r | 0.04586 | 0.44298* | -0.08607 | -0.26015* | -0.1062 | -0.046 | -0.29491* |
|  | *p*-value | 0.73248 | 0.0004973 | 0.51687 | 0.0466 | 0.42341 | 0.73404 | 0.02336 |
| 7-Methylguanine | r | 0.12702 | 0.45827* | -0.03314 | -0.27796* | -0.03051 | 0.04279 | -0.22982 |
|  | *p*-value | 0.34204 | 0.0002974 | 0.80321 | 0.03304 | 0.81856 | 0.75199 | 0.07994 |
| Aspartyl-Histidine | r | 0.15564 | 0.03539 | -0.02668 | -0.20211 | 0.20356 | 0.15233 | 0.00175 |
|  | *p*-value | 0.24335 | 0.792 | 0.841 | 0.12475 | 0.12202 | 0.25797 | 0.98948 |
| N-Isobutyryl-L-cysteine | r | 0.31018* | 0.04525 | -0.02449 | 0.0817 | 0.28307* | 0.27177* | 0.0002046 |
|  | *p*-value | 0.01781 | 0.73592 | 0.85392 | 0.53846 | 0.02982 | 0.04086 | 0.99877 |
| MG(0:0/16:0/0:0) | r | -0.15284 | -0.06376 | -0.0327 | 0.10632 | -0.12554 | -0.0904 | 0.07712 |
|  | *p*-value | 0.25204 | 0.63444 | 0.80576 | 0.42287 | 0.34341 | 0.50363 | 0.56151 |
| PG(16:0/20:3(5Z,8Z,11Z)) | r | -0.11397 | -0.38636* | 0.0004676 | 0.22261 | -0.00792 | -0.02997 | 0.1814 |
|  | *p*-value | 0.39429 | 0.00274 | 0.9972 | 0.09014 | 0.95251 | 0.82484 | 0.16914 |
| Fucα1-2Galβ1-4Glcβ-Sp | r | 0.44465* | 0.34145* | 0.17179 | 0.00374 | 0.37166* | 0.41878* | 0.01622 |
|  | *p*-value | 0.0004707 | 0.00871 | 0.19325 | 0.97756 | 0.00375 | 0.00119 | 0.90296 |
| Glu Glu Thr Val Tyr | r | 0.4312* | 0.35168* | 0.14908 | -0.02348 | 0.33096* | 0.39824* | -0.02072 |
|  | *p*-value | 0.0007269 | 0.00679 | 0.25977 | 0.85989 | 0.01046 | 0.00215 | 0.87622 |
| L-Glutamic acid 5-phosphate | r | -0.02779 | 0.09331 | 0.06503 | 0.34761* | -0.04862 | -0.04165 | -0.13549 |
|  | *p*-value | 0.83594 | 0.48602 | 0.62461 | 0.00698 | 0.71459 | 0.75837 | 0.30624 |
| Asp Ala Pro Ser | r | -0.08455 | -0.42962* | -0.04404 | 0.09413 | -0.01758 | 0.03847 | 0.17059 |
|  | *p*-value | 0.52804 | 0.0007639 | 0.74047 | 0.47826 | 0.89485 | 0.77632 | 0.19644 |
| (11Z)-8,18-methano-retinal | r | -0.06236 | -0.3947* | -0.14692 | 0.01652 | -0.04385 | 0.03666 | 0.09866 |
|  | *p*-value | 0.64193 | 0.00217 | 0.26683 | 0.90116 | 0.74157 | 0.78662 | 0.45721 |

IG: Immature Granulocyte, NRBC: Nucleated Red Blood Cell, Significance * indicates *P* < 0.05

Supplementary Table S5. Spearman Correlation analysis of the key differential metabolites and immune cell profiles in full-term neonates

| Metabolite | Parameter | Neutrophil Percentage | Neutrophil Absolute Count | Lymphocyte Percentage | Monocyte Percentage | Eosinophil Percentage | Basophil Percentage |
| --- | --- | --- | --- | --- | --- | --- | --- |
| 3,5-dihydroxy-4-(sulfooxy)benzoic acid | r | 0.17931 | 0.17475 | -0.20007 | 0.21772 | -0.32825* | 0.42315* |
|  | *p*-value | 0.17042 | 0.18175 | 0.12535 | 0.09471 | 0.01045 | 0.0007557 |
| 1-Methylguanine | r | 0.23647 | 0.24968 | -0.28235* | 0.30792* | -0.26714* | 0.10499 |
|  | *p*-value | 0.0689 | 0.05436 | 0.02883 | 0.01669 | 0.03908 | 0.42469 |
| 7-Methylguanine | r | 0.18512 | 0.21801 | -0.23656 | 0.31059* | -0.21232 | 0.12728 |
|  | *p*-value | 0.15676 | 0.09426 | 0.0688 | 0.01572 | 0.10339 | 0.33249 |
| Aspartyl-Histidine | r | 0.02982 | -0.03659 | -0.03318 | 0.09315 | -0.11871 | -0.21135 |
|  | *p*-value | 0.82109 | 0.78133 | 0.8013 | 0.479 | 0.36632 | 0.10501 |
| N-Isobutyryl-L-cysteine | r | -0.000111 | 0.02342 | 0.01745 | 0.17096 | -0.21446 | 0.18736 |
|  | *p*-value | 0.99933 | 0.859 | 0.89472 | 0.19154 | 0.09988 | 0.15172 |
| MG(0:0/16:0/0:0) | r | -0.03087 | 0.05096 | 0.03462 | -0.06246 | 0.167 | 0.02915 |
|  | *p*-value | 0.81486 | 0.699 | 0.79284 | 0.63541 | 0.20219 | 0.82501 |
| PG(16:0/20:3(5Z,8Z,11Z)) | r | -0.02882 | -0.05527 | 0.08667 | -0.13802 | 0.135 | -0.05979 |
|  | *p*-value | 0.827 | 0.67492 | 0.51024 | 2.93E-01 | 0.30374 | 0.64995 |
| Fucα1-2Galβ1-4Glcβ-Sp | r | 0.22861 | 0.3748* | -0.24873 | 0.37186* | -0.35788* | 0.26678* |
|  | *p*-value | 0.07894 | 0.00317 | 0.05532 | 3.44E-03 | 0.00499 | 0.03935 |
| Glu Glu Thr Val Tyr | r | 0.234 | 0.35744* | -0.24662 | 0.3226* | -0.33375* | 0.23341 |
|  | *p*-value | 0.07194 | 0.00505 | 0.05749 | 1.19E-02 | 0.00916 | 0.07268 |
| L-Glutamic acid 5-phosphate | r | -0.11632 | -0.13371 | 0.11918 | -0.20696 | 0.30731* | -0.25671* |
|  | *p*-value | 0.37613 | 0.30845 | 0.36441 | 1.13E-01 | 0.01692 | 0.04771 |
| Asp Ala Pro Ser | r | -0.14035 | -0.096 | 0.12885 | -0.11472 | 0.26539* | -0.11374 |
|  | *p*-value | 0.28479 | 0.4656 | 0.3265 | 0.38275 | 0.04043 | 0.38689 |
| (11Z)-8,18-methano-retinal | r | 0.1737 | 0.21631 | -0.19146 | 0.19881 | -0.17598 | 0.05492 |
|  | *p*-value | 0.18442 | 0.09691 | 0.1428 | 0.1278 | 0.17863 | 0.67686 |

| Metabolite | Parameter | Immature Granulocyte Percentage | Nucleated Red Blood Cell Percentage | Lymphocyte Absolute Count | Eosinophil Absolute Count | Basophil Absolute Count | Immature Granulocyte Absolute Count | Monocyte Absolute Count |
| --- | --- | --- | --- | --- | --- | --- | --- | --- |
| 3,5-dihydroxy-4-(sulfooxy)benzoic acid | r | 0.45951* | 0.27867* | -0.04302 | -0.27978* | 0.43481* | 0.42472* | 0.20492 |
|  | *p*-value | 0.0003235 | 0.03581 | 0.74413 | 0.03039 | 0.0005174 | 0.0009914 | 0.11627 |
| 1-Methylguanine | r | 0.44031* | 0.30508* | -0.13284 | -0.1574 | 0.22248 | 0.45115* | 0.37266* |
|  | *p*-value | 0.0006092 | 0.02102 | 0.31161 | 0.22972 | 0.08754 | 0.0004281 | 0.00336 |
| 7-Methylguanine | r | 0.41572* | 0.2892* | -0.12462 | -0.10954 | 0.22594 | 0.42634* | 0.35768* |
|  | *p*-value | 0.0013 | 0.02912 | 0.34278 | 0.40477 | 0.08259 | 0.0009434 | 0.00502 |
| Aspartyl-Histidine | r | 0.11483 | 0.18115 | -0.06637 | -0.16716 | -0.13771 | 0.11748 | 0.04869 |
|  | *p*-value | 0.39502 | 0.17749 | 0.61439 | 0.20176 | 0.29407 | 0.38412 | 0.71182 |
| N-Isobutyryl-L-cysteine | r | 0.05485 | -0.02559 | 0.05664 | -0.24012 | 0.18989 | 0.04303 | 0.06997 |
|  | *p*-value | 0.68529 | 0.85016 | 0.66731 | 0.06461 | 0.14617 | 0.75062 | 0.59523 |
| MG(0:0/16:0/0:0) | r | -0.02141 | 0.16731 | 0.1262 | 0.21424 | 0.04866 | -0.01365 | -0.03966 |
|  | *p*-value | 0.8744 | 0.2135 | 0.33663 | 0.10023 | 0.71197 | 0.91972 | 0.76354 |
| PG(16:0/20:3(5Z,8Z,11Z)) | r | -0.06611 | 0.19448 | 0.06178 | 0.12071 | -0.07717 | -0.08152 | -0.15771 |
|  | *p*-value | 0.62514 | 0.14715 | 0.63912 | 0.35824 | 0.55785 | 0.54662 | 0.22881 |
| Fucα1-2Galβ1-4Glcβ-Sp | r | 0.48033* | 0.20571 | -0.00514 | -0.25232 | 0.36614* | 0.5377* | 0.46448* |
|  | *p*-value | 0.000156 | 0.12475 | 0.9689 | 0.05178 | 0.00401 | 1.609E-05 | 0.0001851 |
| Glu Glu Thr Val Tyr | r | 0.45633* | 0.22443 | -0.02001 | -0.22916 | 0.33081* | 0.51234* | 0.42816* |
|  | *p*-value | 0.0003602 | 0.09328 | 0.87938 | 0.07819 | 0.00983 | 4.619E-05 | 0.0006433 |
| L-Glutamic acid 5-phosphate | r | -0.27312* | -0.04318 | 0.03013 | 0.28478* | -0.23642 | -0.30455* | -0.21898 |
|  | *p*-value | 0.03982 | 0.74977 | 0.81926 | 0.02743 | 0.06896 | 0.02126 | 0.09276 |
| Asp Ala Pro Ser | r | -0.33372* | -0.40591* | 0.07009 | 0.28648* | -0.118 | -0.32589* | -0.09743 |
|  | *p*-value | 0.01118 | 0.00173 | 0.59462 | 0.02648 | 0.36921 | 0.01337 | 0.45895 |
| (11Z)-8,18-methano-retinal | r | 0.01411 | -0.31277* | -0.05367 | -0.10009 | 0.16921 | 0.05344 | 0.27476* |
|  | *p*-value | 0.91703 | 0.01784 | 0.68383 | 0.44673 | 0.19621 | 0.69299 | 0.03362 |

Supplementary Table S6. Pathway Analysis of Preterm Neonates at 24 and 48 h. Postpartum

| **Pathways** | **Hits** | ***P*-value** | **-lg(*P*)** | **Impact** |
| --- | --- | --- | --- | --- |
| Citrate cycle (TCA cycle) | 4 | 0.00042 | 3.3767 | 0.22373 |
| Alanine, aspartate and glutamate metabolism | 4 | 0.001596 | 2.797 | 0.13702 |
| Arginine biosynthesis | 3 | 0.001972 | 2.7051 | 0.07614 |
| Phenylalanine, tyrosine and tryptophan biosynthesis | 2 | 0.002056 | 2.687 | 1 |
| Arginine and proline metabolism | 4 | 0.004127 | 2.3843 | 0.18953 |
| Valine, leucine and isoleucine biosynthesis | 2 | 0.009149 | 2.0386 | 0 |
| Phenylalanine metabolism | 2 | 0.009149 | 2.0386 | 0.35714 |
| Ascorbate and aldarate metabolism | 2 | 0.011624 | 1.9346 | 0.52381 |
| Glyoxylate and dicarboxylate metabolism | 3 | 0.021319 | 1.6712 | 0.07408 |
| Butanoate metabolism | 2 | 0.031583 | 1.5005 | 0.03175 |

Supplementary Table S7. Metabolites validated by NMR Spectroscopy

| No. | Metabolite | ppm | *P* | FC | logFC | VIP | Trend  P/F |
| --- | --- | --- | --- | --- | --- | --- | --- |
| 1 | L-Glutamic acid | 3.73 | 0.009009 | 0.984053 | -0.02319 | 1.121068 | down |
| 2 | Homocysteine | 3.89 | 0.006504 | 0.952813 | -0.06973 | 1.291906 | down |
| 3 | 2-Hydroxy-3-methylbutyric acid | 1.48 | 0.006597 | 1.06317 | 0.088372 | 1.318383 | up |
| 4 | Choline | 3.19 | 0.0319 | 1.02109 | 0.03011 | 1.2254 | up |
| 5 | Citrate | 2.65 | 0.001559 | 1.32669 | 0.407831 | 1.77717 | up |
| 6 | Fumaric acid | 6.55 | 0.0081 | 0.7411 | -0.4321 | 1.4736 | down |
| 7 | Phenylalanine | 7.34 | 0.011211 | 1.21739 | 0.283791 | 1.18586 | up |
| 8 | L-Tryptophan | 7.18 | 0.005086 | 2.83864 | 1.5052 | 1.45205 | up |

# Supplementary Figures


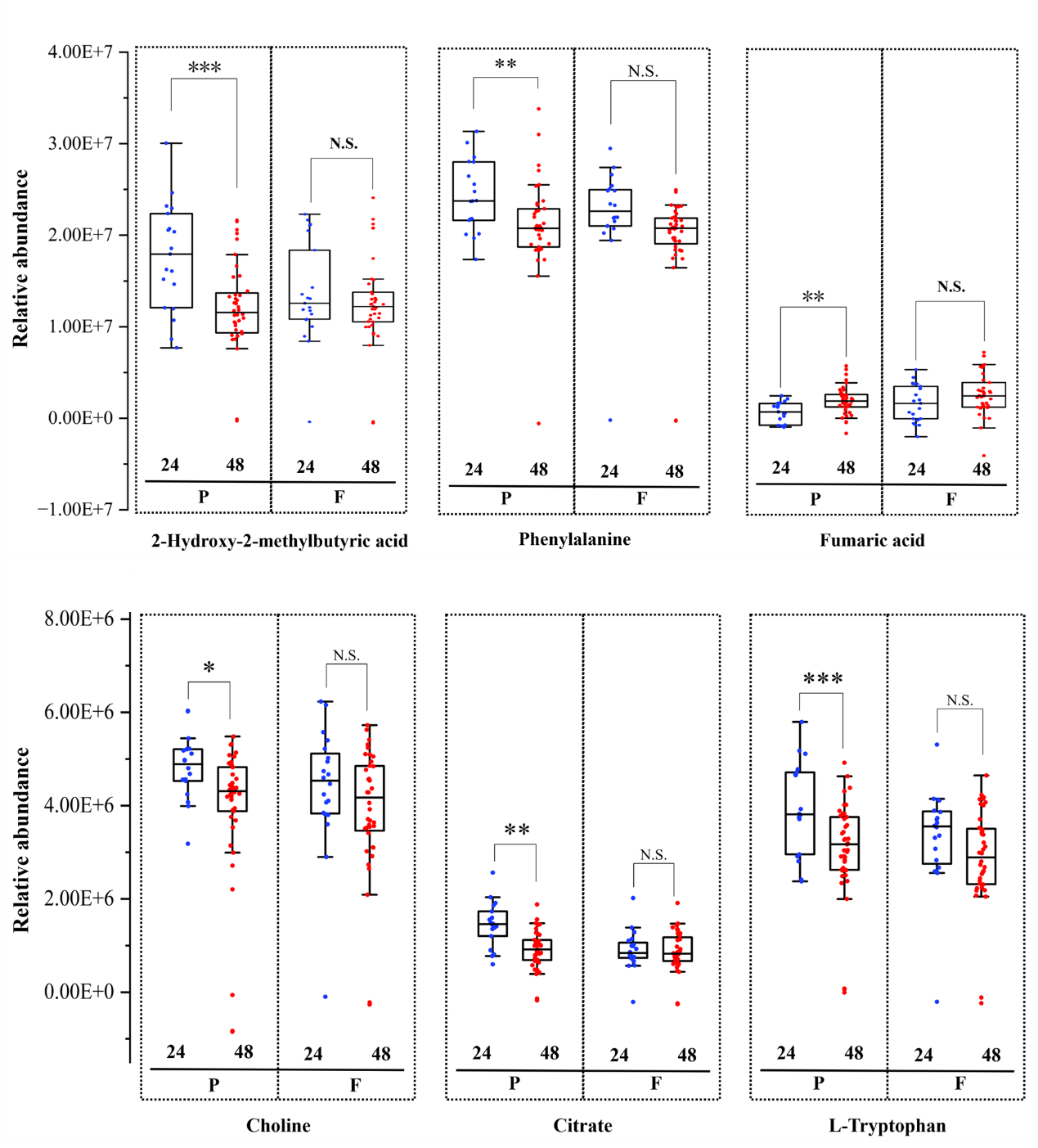


Supplementary Figure S1. Boxplot of six validated metabolites between preterm and full-term neonates at 24 h and 48 h postpartum. The intensity of these metabolites was determined using UPLC-MS based metabolomics.


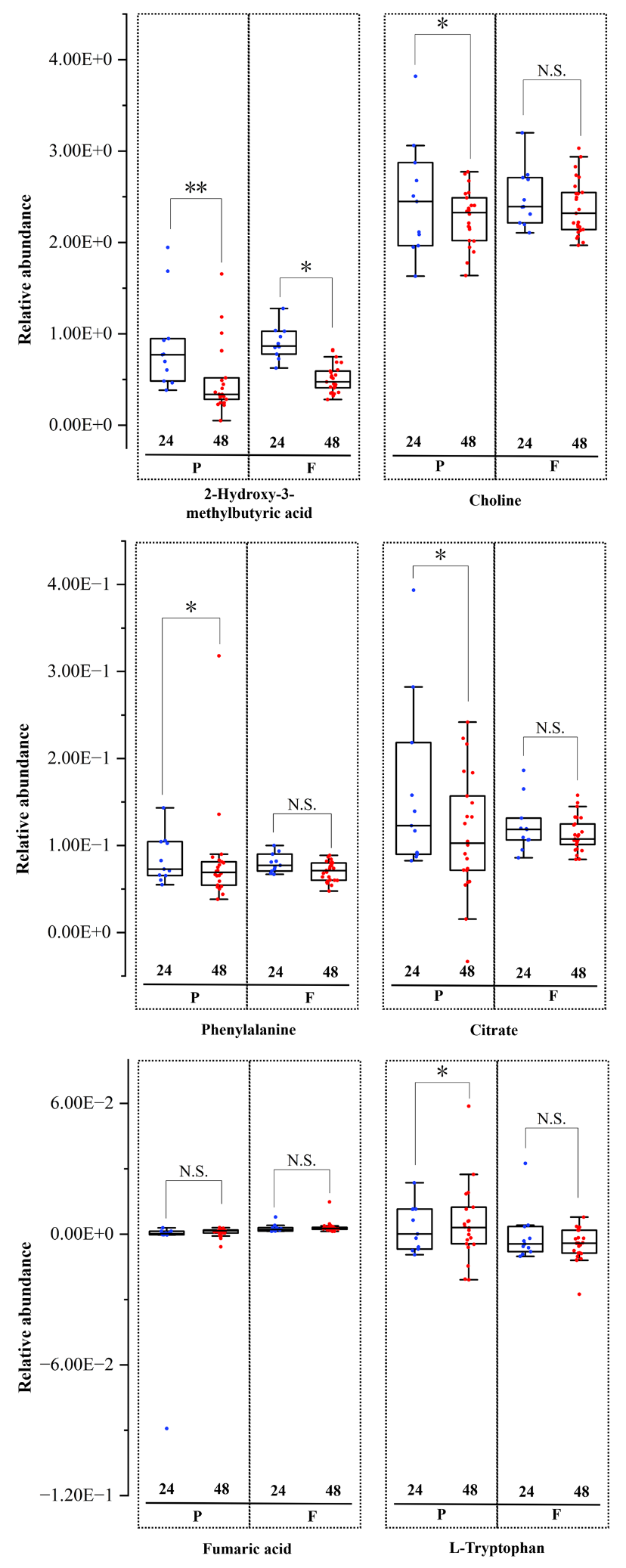


Supplementary Figure S2. Boxplot of six validated metabolites between preterm and full-term neonates at 24 h and 48 h postpartum. The relative abundance of selected metabolites was measured using NMR-based metabolomics.
